# Identification and Characterization of Novel Renal Sensory Receptors

**DOI:** 10.1371/journal.pone.0111053

**Published:** 2014-10-23

**Authors:** Premraj Rajkumar, William H. Aisenberg, Omar W. Acres, Ryan J. Protzko, Jennifer L. Pluznick

**Affiliations:** Department of Physiology, Johns Hopkins University School of Medicine, Baltimore, Maryland, United States of America; Monell Chemical Senses Center, United States of America

## Abstract

Recent studies have highlighted the important roles that “sensory” receptors (olfactory receptors, taste receptors, and orphan “GPR” receptors) play in a variety of tissues, including the kidney. Although several studies have identified important roles that individual sensory receptors play in the kidney, there has not been a systematic analysis of the renal repertoire of sensory receptors. In this study, we identify novel renal sensory receptors belonging to the GPR (n = 76), olfactory receptor (n = 6), and taste receptor (n = 11) gene families. A variety of reverse transcriptase (RT)- PCR screening strategies were used to identify novel renal sensory receptors, which were subsequently confirmed using gene-specific primers. The tissue-specific distribution of these receptors was determined, and the novel renal ORs were cloned from whole mouse kidney. Renal ORs that trafficked properly *in vitro* were screened for potential ligands using a dual-luciferase ligand screen, and novel ligands were identified for Olfr691. These studies demonstrate that multiple sensory receptors are expressed in the kidney beyond those previously identified. These results greatly expand the known repertoire of renal sensory receptors. Importantly, the mRNA of many of the receptors identified in this study are expressed highly in the kidney (comparable to well-known and extensively studied renal GPCRs), and in future studies it will be important to elucidate the roles that these novel renal receptors play in renal physiology.

## Introduction

A recent paradigm in sensory physiology suggests that several classes of understudied receptors (olfactory receptors (ORs), taste receptors, and orphan G-protein coupled receptors (GPRs)) play key roles in non-sensory tissues, where they serve as selective and sensitive chemoreceptors [1], [2], [3], [4], [5], [6], [7], [8], [9], [10], [11], [12], [13]. For example, ORs are expressed in a variety of non-olfactory tissues (including sperm, muscle, brain, and liver) [3], [11], [14], [15] and it has been shown that an OR in the spermatozoa of both humans and mice functions as a chemosensor to help guide the sperm towards the egg [11]. In addition, sweet taste receptors are found in the bladder [2], sour taste receptors facilitate pH sensing in the cerebrospinal fluid [5], bitter taste receptors mediate both bronchodilation and ciliary beat frequency in airways [1], [10], and GPR receptors play important roles in whole-animal physiology as sensors of metabolites [4], [6], [9], [12], [13], [16]. Ligands for these receptors are often generated by metabolic pathways or other physiological processes [5], [12], [17], indicating that known metabolites may have additional (and as-of-yet unknown) signaling functions [4], [12], [17].

We and others have recently demonstrated that the olfactory and GPR signaling pathways play a role in the kidney [7], [8], [12], [13], [18]. We also reported, using a degenerate PCR screen [19], [20], that 6 individual ORs are expressed in mouse kidney by RT-PCR [8]. However, the expression of specific sensory receptors in the kidney and their potential roles is understudied, and the full complement of renal sensory receptors is unknown [16]. The OR gene family alone consists of ∼1000 genes in the mouse, and despite being the largest gene family in the genome it is largely unexplored in the kidney. In addition, although taste receptors have been shown to play important roles in several tissues outside the tongue [1], [2], [10], [21], [22], [23], taste receptor expression in the kidney had not been previously examined. In order to categorize and identify which sensory receptors are present in the murine kidney, we employed several strategies. First, to identify novel renal GPRs, we employed a real-time RT-PCR screen for detection of mouse GPCR transcripts within a mouse kidney cDNA and determined their relative levels of expression. In order to identify whether additional renal ORs (beyond the 6 reported previously [8]) are expressed in the kidney, we performed several small-scale directed RT-PCR screens. Finally, we performed a directed RT-PCR screen for all known murine taste receptors. Together, our study identified 76 novel GPRs, 6 novel ORs, and 11 novel taste receptors expressed in the murine kidney. Subsequently, for a subset of these receptors we analyzed the tissue distribution patterns outside of the kidney, and cloned and studied the receptors *in vitro*.

## Materials and Methods

### RT-PCR

This study was carried out with mice that were housed and treated in accordance with policies and protocol (M013M109) approved by the Johns Hopkins University Animal Care and Use Committee (ACUC), as well as the National Institutes of Health principles and guidelines for the Care and Use of Laboratory Animals. Mice were asphyxiated with CO2 and the tissues required for RNA isolation were quickly removed and stored until future use. C57Bl/6 (Charles River) male mice were asphyxiated with CO_2_ and tissues (tongue, colon, heart, liver, lung, skeletal muscle, small intestine, stomach, kidney and testes) were quickly removed and stored in RNALater (Qiagen) until further use. All efforts were taken to minimize any suffering. RNA was isolated from the tissues using TRIzol reagent (Invitrogen) and samples were further processed using the RNeasy RNA clean-up protocol with on-column DNAase digestion (Qiagen). Tissue specific cDNA was synthesized from 1 µg of purified RNA by reverse transcription (RT; iScript cDNA Synthesis Kit, BioRad). Mock-reverse transcription controls were also prepared from each tissue by omitting the iScript reverse transcriptase enzyme (replaced with an equal volume of water) in reaction mixtures.

PCR was performed using HotStarTaq Master Mix (Qiagen) following standard thermocycling conditions. Murine PCR gene specific primer (GSP) sets were designed using the NCBI Primer Blast PCR primer designer tool for a total of 40 ORs, selected as described in the results section. The nucleotide sequences of the GSP sets along with the expected size band for taste receptors and GPCRs are also listed in Table S1. Prior to screening kidney for novel receptors, we tested our primers and optimized PCR cycling conditions by using either tongue RT (taste) or genomic DNA (ORs). Mock RT reactions were run in parallel with all RT reactions, and all PCR amplicons were sequenced to confirm identity. All RT-PCR products were sequenced to confirm identity.

### Taqman array GPCR screen

To identify novel GPRs and determine their relative expression levels in the kidney, we performed an unbiased screen of whole kidney tissue cDNA using the Taqman array mouse GPCR panel (Applied Biosystems, catalog # 4378703) according to the manufacturers protocol. Briefly, two C57Bl/6 mice (one male & one female) were asphyxiated with CO_2_ and their kidneys were quickly removed and stored. RNA was isolated from the left kidney of both mice using TRIzol reagent (Invitrogen) and 2ug of RNA per reaction was used to synthesize cDNA using the High Capacity RNA-to-cDNA Kit (AB). Each reservoir in the Taqman array microfluidic card was filled with 1000 ng of cDNA per reservoir and the array cards were run on the AB 7900HT Fast RT-PCR system and analyzed using the SDS2.4 software. Each mouse kidney cDNA was screened on 2 chips, for a total of 4 chips. The screen targeted 380 GPCRs including retinal receptors, small molecule receptors, and chemokine receptors in addition to other ‘classic -endogenous’ genes as controls. From the obtained Ct measurements, we calculated ΔCt values of each receptor by normalizing to beta-actin, and further estimated standard deviation (S.D.).

### Surface Immunofluorescence

Full-length coding sequences of mouse Olfr 31, 99, 545, 691, 693 and 1426 were cloned by PCR from mouse kidney RT into a mammalian expression vector, pME18S, with N-terminal Flag and Rho sequences (kind gift from Kazushige Touhara, Univ. of Toyko [24] and Stuart Firestein, Columbia University) between EcoRI and XhoI cloning sites. We also cloned another set of constructs for each OR with a Lucy tag [25] at the N-terminus in addition to Flag and Rho tags. OR constructs were transiently expressed in HEK293T cells with and without chaperone RTP1S (Lipofectamine 2000, Invitrogen). The trafficking of Flag-Rho-tagged/Lucy-Flag-Rho-tagged ORs (+/− RTP1S/Ric8b) in transfected cells was assayed using a surface immunocytochemistry staining procedure as previously described [25], [26], in which a rabbit polyclonal anti-Flag antibody (Sigma) was used in live cells at 4°C. Subsequently, the cells were fixed with 4% PFA, permeabilized using 0.3% triton-X 100 and then exposed to a mouse monoclonal anti-Flag antibody to label internal receptor (Sigma). Fluorescent secondary antibodies (AlexaFluor, Invitrogen) were used to localize the Flag-tagged ORs to the membrane surface or the cytosol of HEK293T cells.

### Luciferase Assay

For ORs that trafficked to the plasma membrane of HEK293T cells (trafficking conditions determined in the surface immunofluorescence assay as described above), we performed an unbiased ligand screen using a dual-luciferase reporter assay (Promega) to identify potential ligands for orphan ORs and to expand the ligand profile of previously deorphanized Olfr691 [26]. Under the conditions (+/− Lucy tag, +/− RTP/Ric8b) that yielded strong surface trafficking for each OR, ORs were transfected into HEK293T cells along with a CREB-dependent luciferase (*Firefly*) and a constitutively expressed luciferase (*Renilla*) [26]. Upon a ligand-OR binding event, a rise in cAMP drives the measurable expression of *Firefly* luciferase, which was normalized to the activity of the *Renilla* luciferase to control for variation in cell number and transfection efficiency. Transfected cells were exposed to potential ligands for 4 hours and their corresponding luciferase values were measured in triplicates, in a semi-automated fashion using a FLUOstar Omega microplate reader (BME Labtech). Cells expressing each OR were tested with a set of odorant mixes (described in [18]) and with an additional mix termed CYCONE (containing cyclopentanone, cyclohexanone, cycloheptanone and cyclooctanone each at a final concentration of 0.3 mM). Any activation to the mixes was further explored by exposing the cells to individual components of the chemical mixture to identify the active ligand of that particular OR. In addition, cells were also exposed to a library of chemicals (listed in Table S2) each tested separately at 500 µM. Following the identification of an active ligand, additional candidate ligands were chosen by varying carbon atomic number (CAN) and functional group type and position. Additionally, a metric for odorant comparison was referenced for identifying multifaceted and structurally diverse analogues of active ligands for testing [27]. EC_50_ values of Olfr691 were calculated based on the response to 10 µM, 50 µM, 100 µM, 0.5 mM, 1 mM, 5 mM and 7.5 mM of active ligands by using Sigmaplot data analysis software. Furthermore, all active ligands for Olfr691 were repeated and confirmed by at least three independent trials.

## Results

### Identification of novel renal murine sensory receptors

#### GPRs

**Figure 1:**
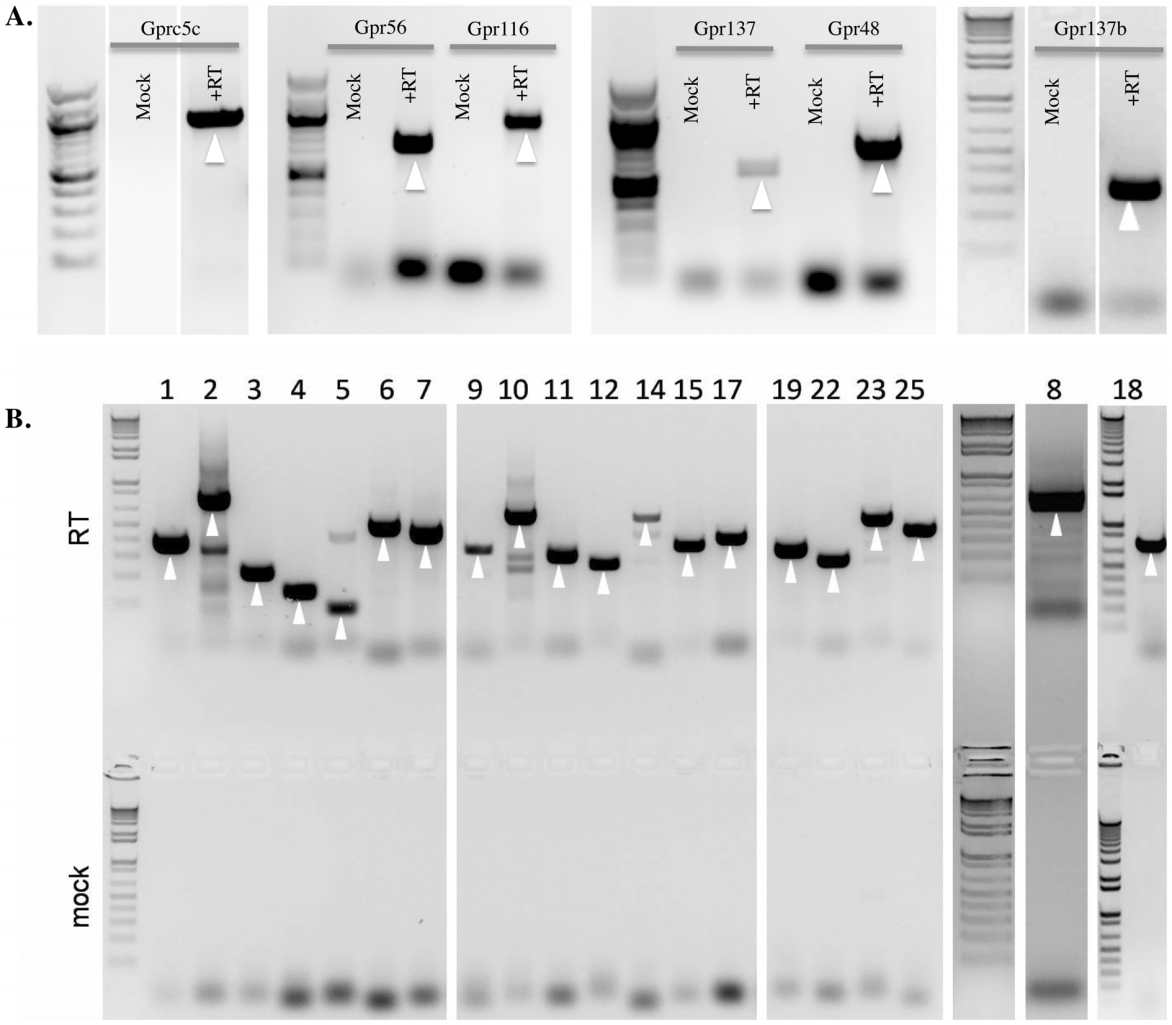
Conventional RT-PCR confirms expression of the six most highly expressed GPRs and top 25 highly expressed transcripts from the TaqMan screen. (**A**) Gprc5c, Gpr56, Gpr116, Gpr137, Gpr48 and Gpr137b were identified as the top six highly expressed GPRs in the mouse kidney based on the TaqMan screen. Mock lanes without RT are negative for all GPRs. The white arrow indicates the expected sized band for each GPR. (**B**) Whole kidney RT and mock RT reaction mixture were screened to validate expression of top 25 highly expressed targets identified from our Taqman array screen: (1)Actb (2) Gapdh (3) Ppia (4) Pgk1 (5) Ubc (6) Calm1 (7) B2 m (8) Pth1r (9) Ywhaz (10) Calm2 (11) Gpr137b (12) Tm7sf3 (14) Agtr1a (15) Sfrp1 (17) Ptger3 (18)Tfrc (19) Hprt (22) Fzd4 (23) Avpr2 (25) Polr2a. All products were sequenced to confirm their identities. The white arrow indicates the expected size band for each receptor.

GPR is the gene name given to orphaned GPCRs; in recent years, as GPRs have been deorphanized, they have been found to play important sensory roles in a variety of tissues [4], [6], [9], [12], [17]. To screen for the expression of GPRs in the kidney, we took advantage of a real time Taqman based mouse GPCR array (Applied Biosystems) which assays the expression of 380 transcripts. This screen is targeted to 380 GPCRs, including 91 GPRs (as well as multiple retinal receptors, small molecule receptors, chemokine receptors and ‘classic -endogenous genes’ as controls). Our analysis focused on the GPRs on this array, as this is a large family of (primarily orphan) receptors which have been shown to play ‘sensory-receptor like’ roles in a variety of tissues [4], [6], [9], [12], [13]. We screened whole kidney tissue from a male & female C57Bl/6 mouse (each kidney was screened on 2 chips, for a total of 4 chips), and calculated average ΔCt values for each receptor by normalizing to beta-actin (Table S3). Based on the average Ct values, we classified the expression level of each receptor in the mouse kidney into one of four categories: high expression (ΔCt ≤7.5), medium expression (7.5≥ ΔCt ≤12.5); low expression (12.5≥ ΔCt ≤20) and null expression (ΔCt ≥20). Among 380 receptors assayed, a total of 30 receptors were highly expressed (average ΔCt ≤7.5) in all four chips, out of which six were GPRs. In addition, a total of 95 receptors had medium levels of expression (7.5≥ ΔCt ≤12.5), out of which 23 were GPRs, and 175 receptors were present with low levels of expression (12.5≥ ΔCt ≤20) of which 51 were GPRs. 80 receptors were found to be not expressed (ΔCt ≥20), of which 11 were GPRs. As a point of reference, GPCRs that are well known to be in the kidney, such as the Angiotensin II 1a receptor (Agtr1a), Arginine vasopressin 2 receptor (Avpr2) and Parathyroid hormone 1 receptor (PTHR1) are present at a high expression level in our array and had an average ΔCt of 6.25±0.24, 7.27±0.17 and 4.38±0.30 respectively. We validated the expression of the top 25 receptors identified through our Taqman array (which includes the top six GPRs: Gpr137, Gpr137b, Gpr56, Gpr48, Gprc5c and Gpr116) by performing ‘conventional’ RT-PCR on the mouse whole kidney cDNA with a separate set of GSP primers, followed by sequencing to confirm identity (Figure 1A, Figure 1B). We did not observe any noticeable differences in the GPR expression levels among male and female kidney.

#### Olfactory receptors

**Figure 2:**
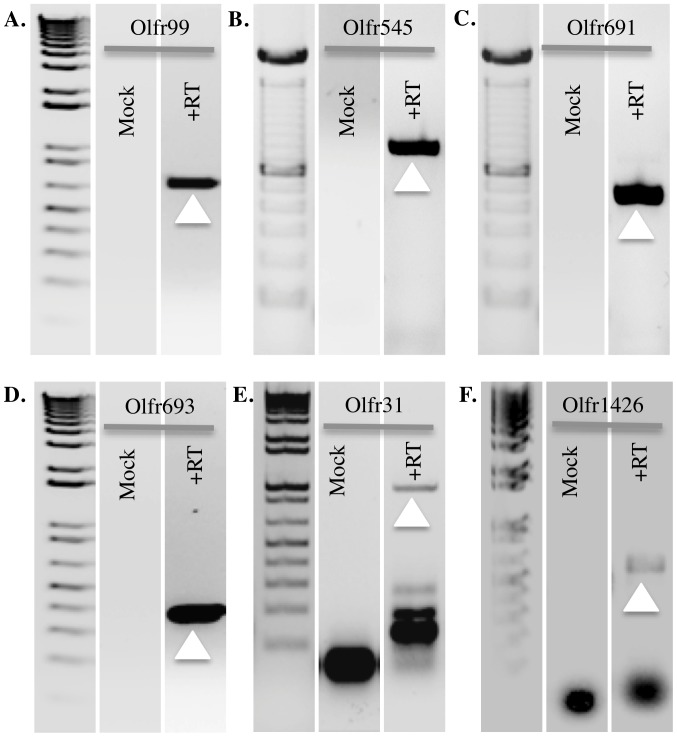
RT-PCR with mouse whole kidney cDNA as template to identify novel renal olfactory receptors. Olfr99 (A), Olfr545 (B), Olfr691 (C), Olfr693 (D), Olfr31(E) and Olfr1426(F) expression is detectible in mouse whole kidney cDNA by PCR and sequencing confirms the identity of amplified products. Mock RT template controls are negative for OR GSP sets and β-actin (not shown). The white arrow indicates the band of the expected size for each olfactory receptor.

In order to identify novel renal olfactory receptors (ORs) in mouse kidney, we undertook an RT-PCR approach. Although most ORs are orphan receptors with no known ligands, a minority of ORs do have identified ligands, and we reasoned that this group of receptors may be advantageous to study if they are expressed ectopically since (at least one) ligand(s) are already known. Therefore, we first performed an RT-PCR screen using primers for murine olfactory receptors that already have reported ligands [4], [6], [27], [28], [29], [30], [31], [32]. The primers (Table S1) were first verified using mouse gDNA (taking advantage of the fact that ORs do not contain introns) as a positive control and to optimize cycling conditions, and then were used on reverse-transcribed kidney cDNA using identical cycling parameters. Of the twenty-nine OR primer sets used, two detected novel renal ORs: Olfr545 (MOR42-1, S50) and Olfr691 (MOR31-6) (Figure 2).

A second RT-PCR screen was performed on mouse kidney cDNA using gene specific primer (GSP) sets directed against ORs which had been previously reported in the literature to be present in renal tissues or cells. First, we assayed for the presence of nine mouse ORs listed by NCBI- Homologene as the corresponding orthologs of rat ORs identified in native rat inner medullary collecting duct (IMCD) cells by a proteomic screen [29] (additional ORs were identified in the original study for which murine orthologs had not been identified, and thus these ORs were not pursued in our study). Bands of the correct size were obtained for two OR primer sets: the murine ortholog of rat Olr1739 (mouse Olfr99), (Figure 2), and the murine ortholog of rat Olr217 (mouse Olfr705). Sequencing confirmed the presence of Olfr99 (MOR156-1), but revealed that the Olfr705 primers had actually amplified the closely related murine OR, Olfr693. We did not observe any chimeric olfactory receptor products in our sequencing results, and subsequent PCR using Olfr693-specific primers confirmed that Olfr693 (aka MOR283-8) is expressed in the kidney (Figure 2). In addition, we were also able to successfully amplify and clone full length Olfr693 receptor from the mouse kidney cDNA. We also screened for the murine homolog of human renal olfactory receptor, OR2T1 [33], and identified that the murine ortholog Olfr31 is present in the whole kidney cDNA (Figure 2). Finally, we also identified that murine Olfr1426, ortholog of a rat OR in the collecting duct and thick ascending limb (M. Knepper, NIH, personal communication), is expressed in the whole kidney cDNA.

#### Taste receptors

**Figure 3:**
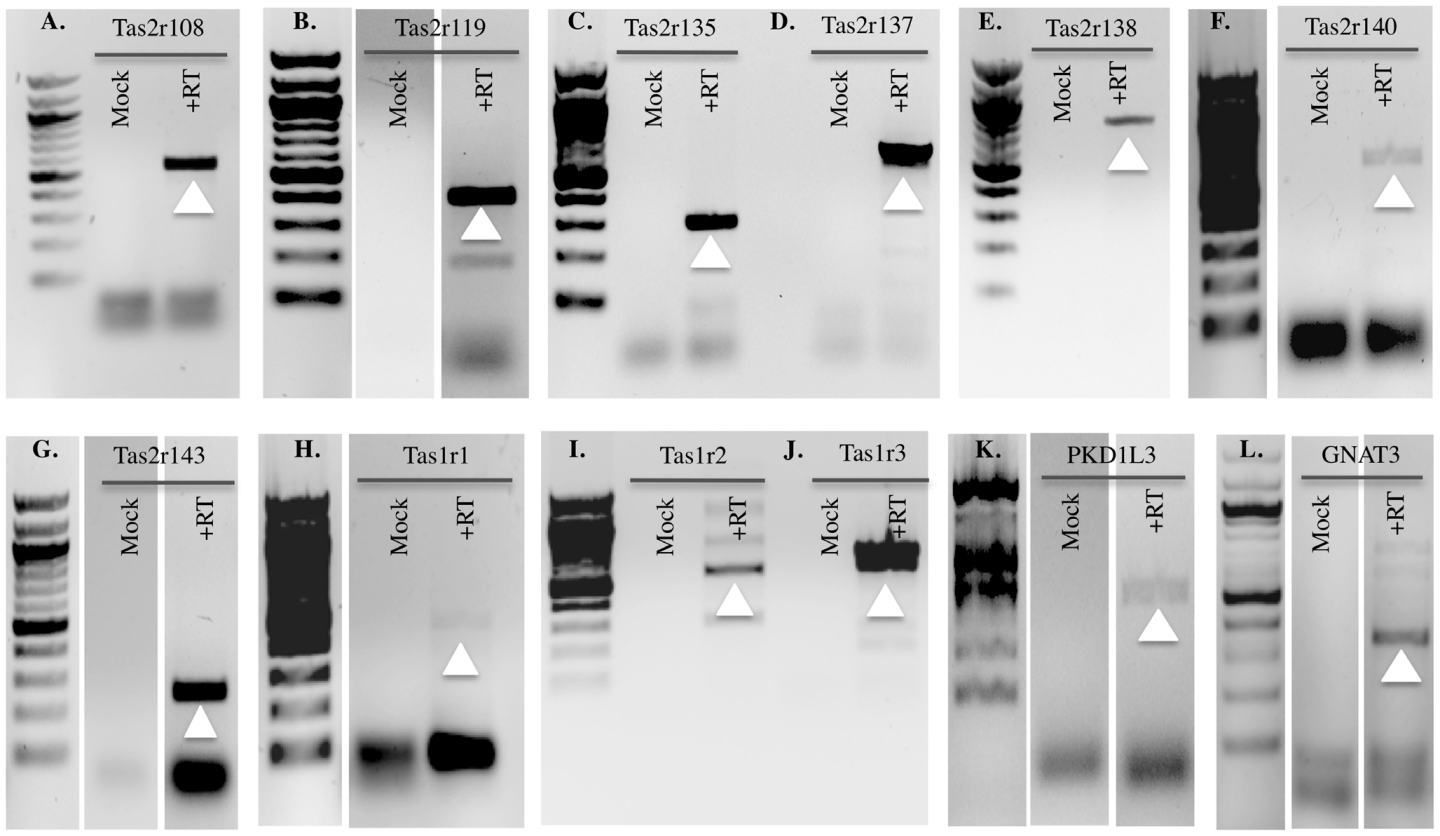
RT-PCR with mouse whole kidney cDNA as template to identify novel renal taste receptors. Tas2r108 (A), Tas2r119 (B), Tas2r135 (C), Tas2r137 (D), Tas2r138 (E), Tas2r140 (F), Tas2r143 (G), Tas1r1 (H), Tas1r2 (I) and Tas1r3 (J) PKD1L3 (K) and G_NAT3_ (L) expression detected in the mouse whole kidney cDNA by RT-PCR and confirmed by sequencing. Mock controls without RT are negative in all the lanes. The white arrow indicates the band of the expected size for each olfactory receptor.

We designed thirty-five GSPs to identify known taste receptors expressed in the kidney using an RT-PCR approach. We used mouse tongue cDNA as the positive control to validate primers (Table S1) and to optimize PCR cycling conditions, and subsequently used the exact cycling conditions on reverse-transcribed kidney cDNA. We identified expression of the three Tas1r receptors, which together mediate both sweet and umami taste (Tas1r2+ Tas1r3 mediate sweet taste, whereas Tas1r1+ Tas1r3 mediate umami taste) [34]. In addition, seven bitter taste receptors (Tas2r108, Tas2r119, Tas2r135, Tas2r137, Tas2r138, Tas2r140 and Tas2r143) and a sour taste receptor, PKD1L3 [35], were identified in the kidney (Figure 3). The salt receptor (ENaC) is already known to be expressed in the kidney where it plays an important role in sodium handling [36], [37], [38]; therefore, we did not include it in our screen. In addition, we also identified expression of G_NAT3_ (the G-protein that mediates taste perception in the tongue) [39] in whole mouse kidney as well (Figure 3L).

### Tissue distribution of renal Sensory Receptors

Ultimately, we are interested in understanding the physiological roles played by these receptors. We were curious, therefore, whether these receptors are expressed ectopically only in the kidney, or if they have wider tissue distributions. To that end, we used an RT-PCR and sequencing approach to assay whether the novel renal sensory receptors we had identified were also found in other tissues. As summarized in Table 1, we found that the expression of these receptors was not limited to the kidneys, and that the tissue distribution profile was unique to each individual receptor. Of the six ORs assayed, Olfr99 had the widest tissue expression profile, present in every tissue that we screened except for skeletal muscle. In addition to the kidneys, Olfr31 and Olfr1426 were expressed only in one another tissue (testes), whereas the remaining ORs were found in at least 3 additional tissues. Every tissue screened except skeletal muscle expressed at least one of the renal ORs, and intriguingly, each of the novel renal ORs was also expressed in the testes (a tissue where ORs have previously been shown to play an important role) [11]. It should be noted that the cDNA from all tissues yielded bands for β-actin.

**Table 1:** Summary of the tissue expression profile of all the novel sensory receptors identified in the mouse whole kidney cDNA.

Receptor	Kidney	Testes	Colon	Heart	Liver	Lung	Skeletal	Small Intestine	Stomach
Olfr31	+	+	−	−	−	−	−	−	−
Olfr99	+	+	+	+	+	+	−	+	+
Olfr545	+	+	−	+	+	−	−	−	−
Olfr691	+	+	+	+	−	+	−	−	+
Olfr693	+	+	−	−	−	+	−	−	+
Olfr1426	+	+	−	−	−	−	−	−	−
Tas2r108	+	+	−	−	−	−	−	−	−
Tas2r119	+	−	−	−	−	−	−	−	−
Tas2r135	+	+	−	+	−	+	−	−	−
Tas2r137	+	−	−	−	−	−	−	−	−
Tas2r138	+	+	−	−	−	−	−	−	−
Tas2r140	+	−	−	−	−	−	−	−	−
Tas2r143	+	−	−	+	−	+	−	−	−
PKD1L3	+	+	−	−	−	−	−	−	−
Tas1r1	+	−	+	−	−	−	−	−	−
Tas1r2	+	−	−	−	−	−	+	−	−
Tas1r3	+	+	−	−	−	+	−	−	−
Gpr56	+	+	−	−	−	−	+	−	+
Gprc5c	+	+	−	−	+	−	+	−	+
Gpr116	+	+	−	−	−	+	+	+	−
Gpr137	+	+	+	−	−	−	−	−	−
Gpr48	+	+	+	−	−	−	−	+	+

A ‘+’ sign indicates expression of the corresponding receptor in our RT-PCR screen whereas a ‘−’ sign indicates absence in that particular tissue. All ‘+’ signs in the table were confirmed by sequencing to confirm identity. In each case, the mock sample without reverse transcriptase during cDNA synthesis was negative.

Of the eleven taste receptors that we assayed by the RT-PCR approach, Tas2r135 had the widest tissue distribution profile, with expression identified in the heart, lung and testes. In contrast, among the tissues we screened, the expression of Tas2r119, Tas2r137 and Tas2r140 was seen only in kidney. The remaining receptors were found in at least one tissue in addition to kidney (Table 1).

We also assayed the tissue distribution of the five most highly-expressed renal GPRs from the TaqMan array (Gprc5c, Gpr48, Gpr56, Gpr116 and Gpr137). These five novel renal GPRs were all found to be expressed in the testes, as well as 1–3 additional tissues (Table 1). All PCR reactions were run along with mock RT controls and amplicons were sequenced to confirm identity.

### Trafficking of newly identified murine renal ORs in HEK293T

In order to understand the function of these receptors in physiology, it is necessary to understand their ligand profiles. Unfortunately, the majority of ORs are orphan receptors with no known ligands. Therefore, using RT-PCR we cloned Olfr99, 545, 691, 693, 31 and 1426 from kidney into expression vectors with N-terminal Flag and Rho tags (+/− Lucy tags; clones were sequenced to confirm identity).

**Figure 4:**
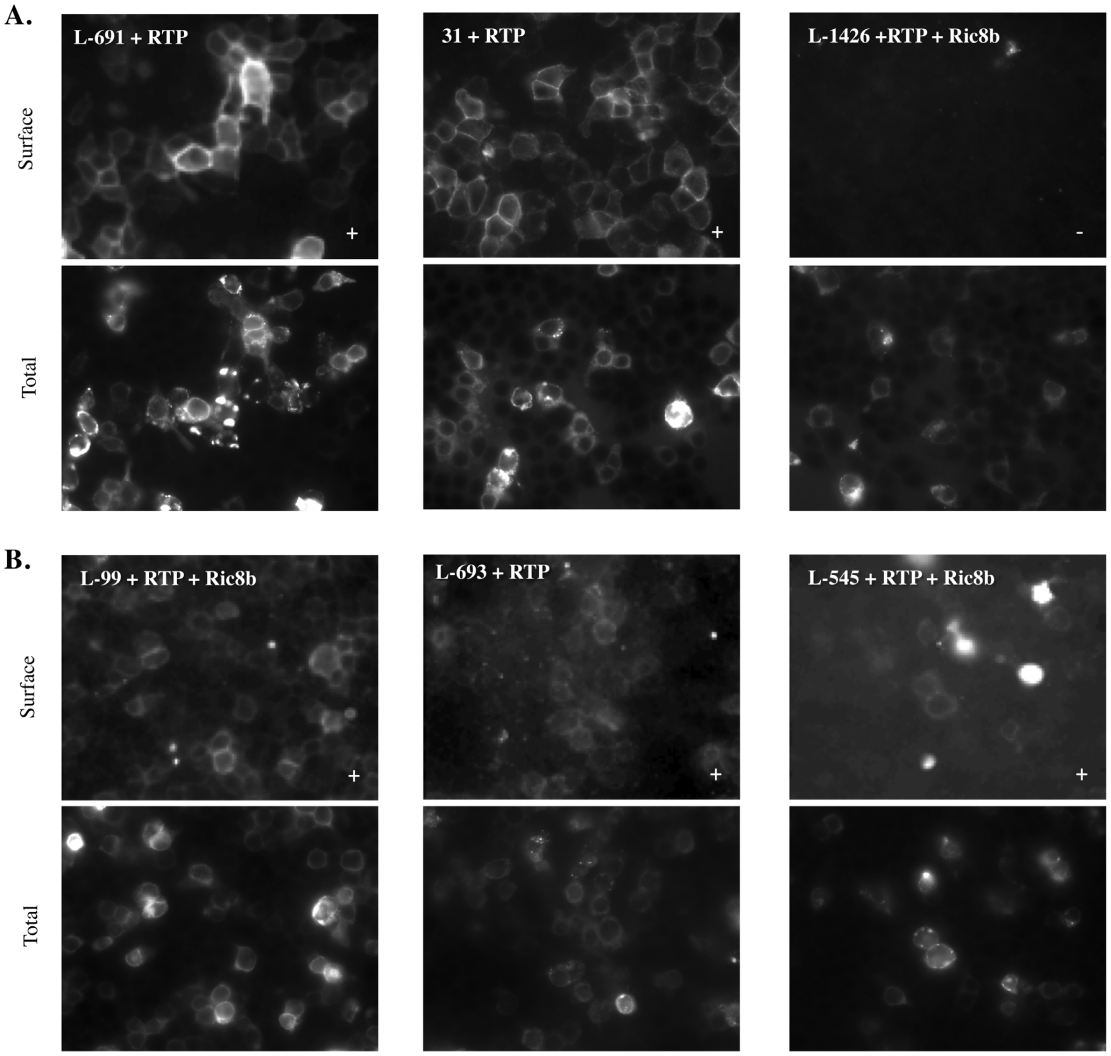
Immunohistochemistry showing surface expression of ORs. Each OR is shown under the experimentally determined condition which allowed for optimized surface expression in HEK293T cells. The surface trafficking conditions vary for each OR and we have published the corresponding conditions for Olfr99, 545, 691 and 693 previously [25]. Briefly, Olfr31 requires co-expression of RTP1S; Olfr691 & Olfr693 require presence of N-terminal Lucy tag along with co-expression of RTP1S; Olfr99, Olfr545 & Olfr1426 requires presence of N-terminal Lucy tag along with co-expression of RTP1S and Ric8b respectively. Olfr31 requires co-expression of RTP1S and Olfr1426 failed to reach the membrane surface at all the tested conditions. HEK293T cells were first stained with a poly-flag antibody (surface) then subsequently permeabilized and stained with a mono-flag antibody (total). The images were taken at equal exposure between all surface and total conditions. Surface images are marked with either a+or – in their lower right-hand corners to indicate the presence or absence of surface expression, respectively. Images in (B) have been enhanced to better display surface expression. Images in (A) are presented as they were taken. Unenhanced images for Figure 4B can be found in Figure S1.

In order to screen an OR for potential ligands, it must be expressed on the cell surface and unfortunately, trafficking of ORs to the cell surface has historically been a problem in the field [30]. Surface expression can sometimes be achieved or enhanced by the concurrent expression of chaperones, most notably receptor transport protein 1 short (RTP1S) [32], [33] or by the use of N-terminal tags (such as Rho [33] or Lucy [25]). We have previously tested and published the conditions under which Olfr99, 545, 691 and 693 reach the cell surface [25]. To determine whether the other novel renal ORs identified here are able to traffic to the cell surface, we used surface immunofluorescence to assay the ability of Flag and Rho (+/− Lucy) tagged ORs to traffic to the surface of HEK293T cells (+/− RTP1S). The optimized condition that facilitates membrane surface trafficking varies for each OR. Briefly, Olfr31 requires co-expression of RTP1S; Olfr691 & Olfr693 require presence of N-terminal Lucy tag along with co-expression of RTP1S; Olfr99 and Olfr545 requires presence of N-terminal Lucy tag along with co-expression of RTP1S and Ric8b (Figure 4). As seen in Figure 4, we observe surface expression for every OR tested with the exception of Olfr1426, which failed to reach the cell surface.

### Ligand profiles

**Figure 5:**
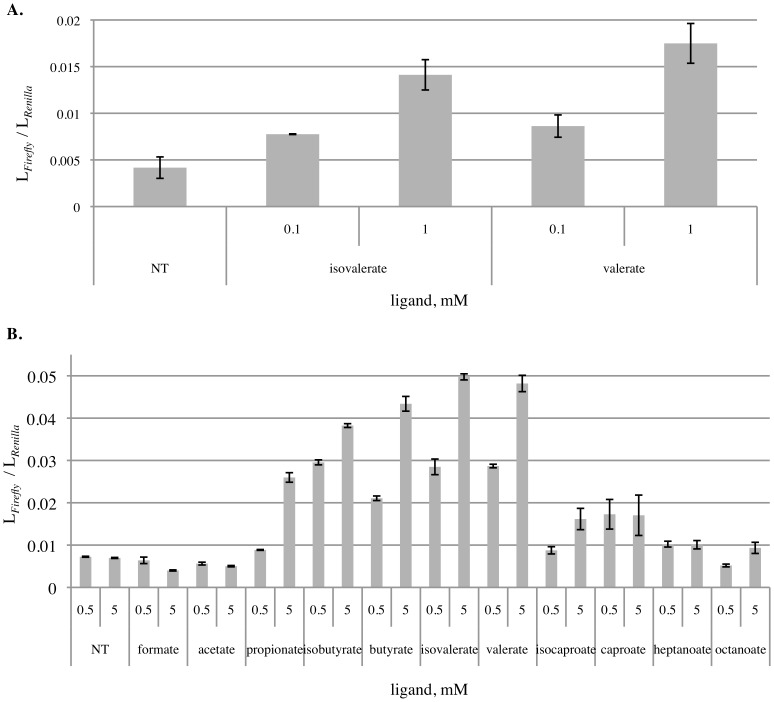
Ligand screening for Olfr691. Olfr691 responds to published short chain fatty acids, isovalerate and valerate, in a dose dependent manner when co-expressed with RTP1S (A). Further ligand screening shows that Olfr691 responds to wide range of saturated short and medium chained fatty acids, from propionate to octanoate, but not including formate and acetate (B). NT represents measurements obtained from non-treated cells (with no ligand) transfected with Olfr691 and RTP1S.

Because Olfr31, 99, 545, 691 and 693 trafficked to the cell surface of HEK 293T cells, we proceeded to examine the ligands of these ORs. Using a cAMP-luciferase reporter assay [26], we tested the response of ORs to an unbiased library of odorant mixes that cover a wide range of odorant space [18], as well as a library of diverse chemicals not biased to a particular olfactory receptor (listed in Table S2). The ligand mixes, the chemical library, and mouse urine all failed to evoke any response from Olfr31, 99, 545 and 693. However, we confirmed previous reports [32] that Olfr691 responds to carboxylic acids valerate and isovalerate in a dose dependent manner (Figure 5A). Previously reported ligands such as pentanal for Olfr691 [32] and sebacic acid for Olfr545 [40] did not induce a response in our luciferase assay.

**Figure 6:**
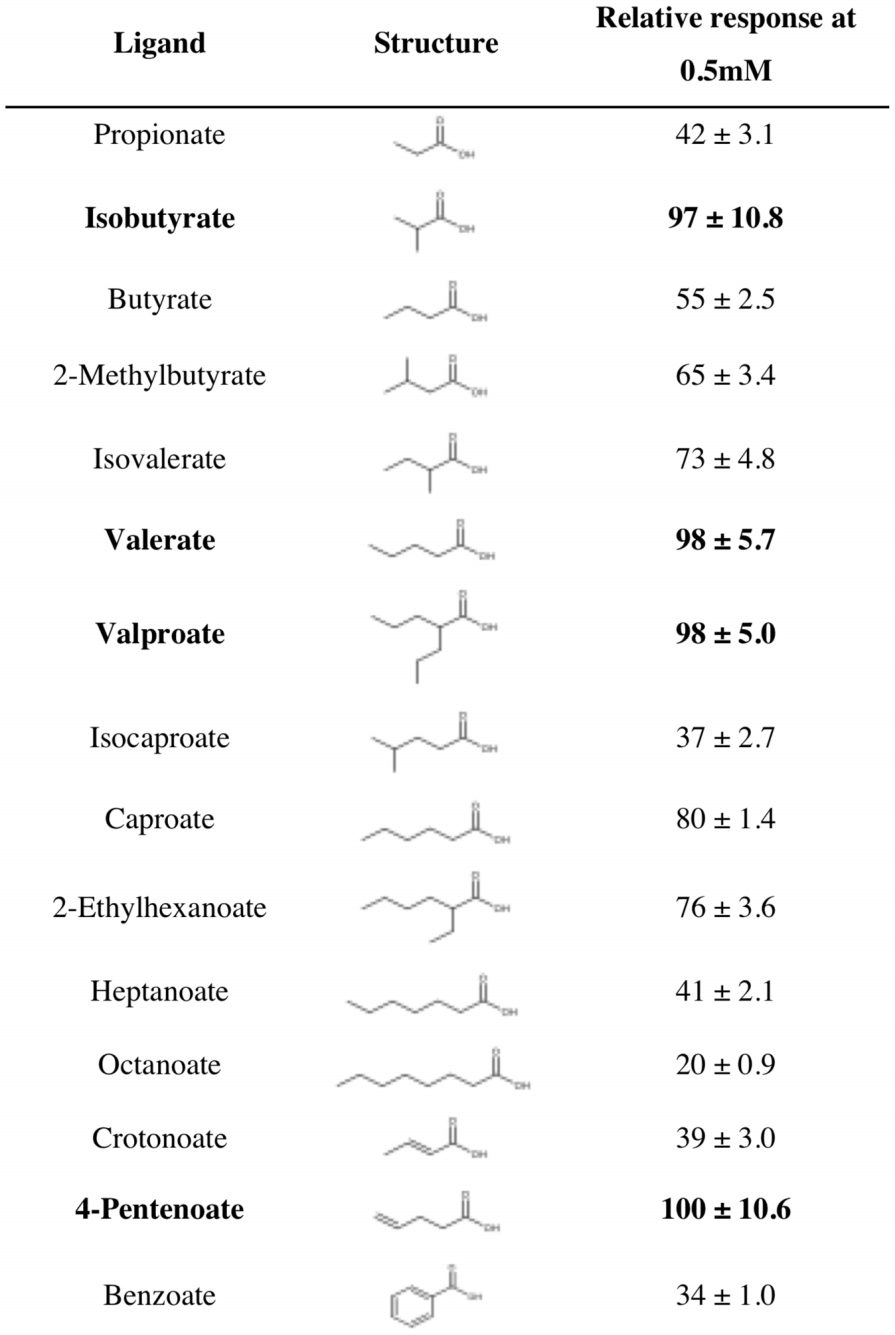
Relative response values at 0.5 mM for Olfr691 ligands. The structures of the ligands are shown in the figure for reference.

**Figure 7:**
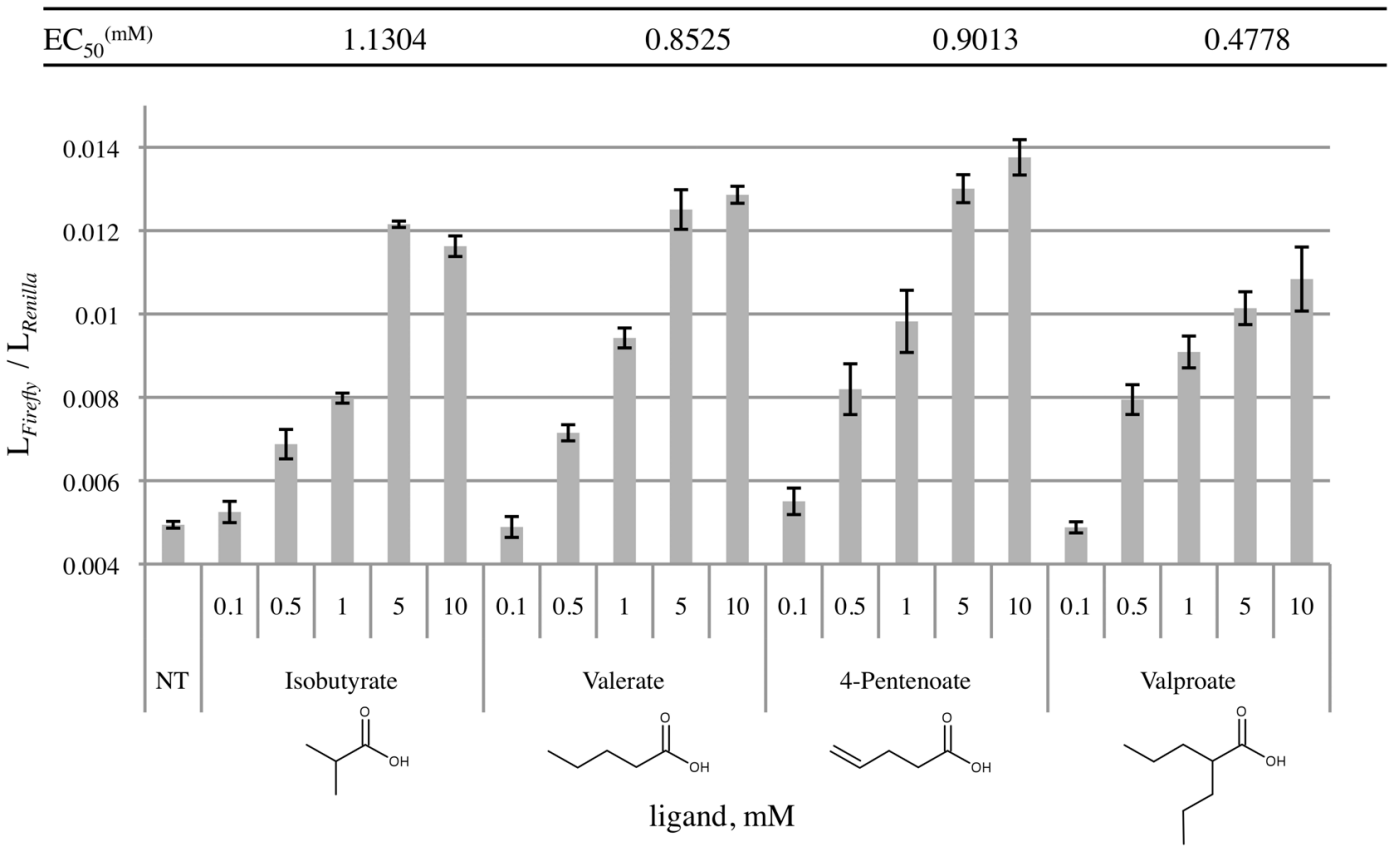
Dose response curves for the novel Olfr691 ligands. Dose response curves show that Olfr691 has the highest affinity for valproate when co-expressed with RTP1S in HEK293T cells, with an EC_50_ value of 0.4778 mM; however 4-pentenoate induced the strongest cAMP responses at all doses when compared to isobutyrate, valerate and valproate. NT represents measurements obtained from non-treated cells (with no ligand) transfected with Olfr691 and RTP1S.

To determine if we could expand upon the known ligands for Olfr691, we then tested Olfr691 using compounds similar to valerate and isovalerate (Figure 5B). We selected ligands using a multidimensional physiochemical metric for odorant prediction which takes into account a variety of molecular characteristics in addition to the traditional values of carbon number and functional group [27]. As summarized in Figure 6, we found that Olfr691 senses a wide range of both short and medium chain fatty acids, binding to carboxylic acids with carbon lengths of three (propionate) to eight (octanoate). Chemical structures for ligands tested are shown in Figure 6 (in their carboxylate form, for simplicity and to reduce space). Olfr691 was not responsive to dicarboxylic acids, amino acids or aldehydes with similar carbon lengths and structures to identified ligands (A complete list of compounds tested for Olfr31, 99, 545, 691 and 693 is shown in Table S2). In this study, we identified thirteen new ligands for Olfr691 in addition to its previously published ligands [32]. Response values in Figure 6 (0.5 mM) have been normalized to the response of Olfr691 to the strongest ligand, 4-pentenoate. Branched chain and alkene analogues of short chain fatty acids and the aromatic carboxylic acid, benzoic acid, as suggested by the physiochemical metric for odorants [12], also induced Olfr691 responses. Detailed dose response curves and EC_50_ values were calculated for four ligands inducing the strongest response at 0.5 mM (Figure 7), shown in bold in Figure 6. An allylic analogue of valerate, 4-Pentenoate, induced the strongest response (Figure 6), whereas valproate had the lowest EC_50_ (0.4778 mM).

## Discussion

Recent studies in the literature have highlighted the important roles that sensory receptors, including ORs, taste receptors and novel GPRs, play in a variety of different tissues [1], [2], [3], [4], [5], [6], [7], [8], [9], [10], [11], [12], [13]. To better understand the roles that sensory receptors play in the kidney, it is first necessary to identify and categorize the full complement of such receptors. To this end, in this study we aimed to identify expression of novel ORs, taste receptors and GPRs in the kidney by using a variety of approaches.

### Identification of Novel Receptors

We previously identified 6 renal ORs using a degenerate OR (dOR) primer screen [19], [20]; however, this approach is biased towards those ORs with the highest levels of expression in a given tissue. In this study, we wanted to determine whether there are any renal ORs beyond the 6 identified previously. Others have successfully used microarrays to detect the expression of ORs in the olfactory epithelium (OE) and elsewhere [41], [42], but ORs in the kidney are expressed in a lower level than in the OE. This low level of expression increases the probability that a microarray may result in false negatives. Therefore, in this study we employed a PCR-based approach using GSPs in order to assay whether additional renal ORs, not detected in our original degenerate OR primer screen, may also be expressed in the kidney. Although not a comprehensive screen, our results clearly show that additional ORs are found in the kidney. In addition to novel renal ORs which have published ligands, we also assayed for renal ORs which had been reported by others to be present in the renal tissues. However, in some cases we found some but not all of the previously reported ORs: for example, previous work on freshly isolated native rat IMCD cells conducted by the Knepper Laboratory at the NIH had identified 19 novel renal ORs [29]. We generated GSP sets against the mouse homologs of these renal rat ORs and detected only Olfr99 and Olfr693 in murine kidney. The discrepancy between our findings is very likely due to the difficulty of identifying OR homologues across species, especially when there are a large number of highly homologous ORs in both species (∼1000 OR genes in mice, and ∼1400 in rats (13; 37)). Therefore, although we only confirmed 2 out of the 19 ORs reported by the Knepper Laboratory, this may represent the limitations of the ability to correctly assign homologues based on sequence similarities. However, the fact that 19 novel ORs were identified by looking at the IMCD alone indicates that a more thorough screen for ORs within the whole kidney is necessary and justified in order to identify the full complement of renal ORs.

It is worth noting that Olfr691 and Olfr31 do have a human homologue listed in NCBI (OR52B2 and OR2T1 respectively), however, there are no human homologs listed for Olfr99, 545, 693 or 1426. Therefore, future studies will be necessary to determine if Olfr99, 545, 693 or 1426 may have functional orthologs in human.

As the taste receptor family is relatively small, we screened for the full complement of taste receptors using 35 gene specific primer (GSP) sets. This direct approach is well suited to screen small families of receptors, as it is cost effective and sensitive to low-level expression receptors. Expression of taste receptors (including the bitter receptors) has been previously identified in non-gustatory tissues [1], [2], [10], [21], [22], [23], [43], however, our study is the first to identify taste receptors in the mouse kidney. From the previous literature on taste receptors, we know that the mouse heteromeric umami (Tas1r1+Tas1r3) and sweet (Tas1r2+Tas1r3) receptors are broadly tuned and that they respond to a variety of L-amino acids and sugars, respectively [34], [44], [45]. Since we detected expression of all three Tas1r subunits (Tas1r1/Tas1r2/Tas1r3) in the mouse kidney, future work is necessary to understand the dimerization characteristics (Tas1r1+Tas1r3 vs. Tas1r2+Tas1r3) of these receptor subunits in the kidney along with their potential renal role towards mediating amino acid and energy homeostasis. Although there is no previous data in the literature regarding taste receptors in the kidney, Tas2r135 and Tas2r143 were previously reported to be expressed in the heart [43], in agreement with our findings.

In addition, because several novel GPRs have been found to play sensory roles in a variety of non-renal tissues, but have not been well studied in the kidney, we undertook a high-throughput approach to assay for the expression of 91 GPRs in the kidney using real-time PCR. In our screen, we detected expression of previously reported GPRs with known cardiovascular and renal functions (C_t_: Gpr30 = 28.67±0.49; Gpr43 = 32.29±0.26; Gpr48 = 23±0.29 and Gpr91 = 23.91±1.04) [6], [12], [46], [47], [48].

Finally, it should be noted that we assayed receptor expression in whole kidney tissue. We cannot rule out that receptors we found to be ‘absent’ are in fact expressed, but only in a small subset of cells (i.e., macula densa, intercalated cells, etc.). In this case, although these receptors may have significant renal roles, they may appear as null expressers in a screen of whole kidney.

### Identification of Novel Ligands

In this study, we screened Olfr31, 99, 545, 691 and 693 in a luciferase assay system to identify their ligands. We identified thirteen novel ligands for Olfr691, but did not identify any ligands for Olfr31, 99, 545 and 693. It is possible that Olfr31, 99, 545, and 693 are narrowly tuned receptors which do not respond to the chemical profiles in our odorant library mixtures [49]. Valerate, isovalerate and pentanal were previously reported [32] as Olfr691 ligands. Of these three, we were able to confirm valerate and isovalerate, but not pentanal. In addition to these previously reported ligands, we now show that Olfr691 is broadly tuned towards carboxylic acid activation, including short and medium chain fatty acids (physiological concentrations are within the range of the ligand concentrations assayed for isovalerate (0.89±0.93 uM [50])). Interestingly, gut bacterial metabolism is the primary physiological source of short chain fatty acids in the bloodstream, with the concentrations reported for propionate varying from 0.1–10 mM [6]. In addition, the response of Olfr691 to valproate is quite intriguing. In clinical trials, patients treated with valproate as an antiepileptic drug have been shown to develop Fanconi syndrome [51], [52], where the renal proximal tubules are affected resulting in an excessive spillage of amino acids, phosphate, glucose, bicarbonate, and uric acid in their urine. In support of this hypothesis, in preliminary studies we observed successful amplification of Olfr691 in cDNA isolated specifically from the S1 and S3 segment of proximal tubule (n = 3). Clearly, future work will need to be done to investigate the relevant *in vivo* renal role of Olfr691.

## Summary

In this study, we have identified expression of novel olfactory receptors, taste receptors and GPRs in the kidney, thereby extending the list of previously known renal sensory receptors. Despite the fact that we screened only part of the OR gene family, and did not screen the trace amine- associated receptor (TAAR) or vomeronasal receptor (VR) families, we were able to identify 93 novel murine sensory receptors, many of which were expressed at high levels by real-time PCR. These data imply that there is a large and robust complement of sensory receptors in the kidney which have not yet been examined in a functional context. Our study is an important first step in identification of novel renal receptors, and future work is now required to localize these receptors within the kidney and to elucidate the physiological role of each receptor.

## Supporting Information

Figure S1 **Unenhanced surface images from** **Figure 4B** **.** Unenhanced images of Olfr99, Olfr693 and Olfr545 in their corresponding conditions that facilitate plasma membrane surface trafficking in HEK293T cells.
(TIFF)

Table S1 **Nucleotide sequences of the primers used to screen cDNA synthesized from mouse whole kidney.** Sequences of both the forward and reverse primers used to screen whole kidney cDNA in our RT-PCR approach along with their expected size bands.
(XLSX)

Table S2 **List of all ligands used to screen Olfr691, Olfr99, Olfr545, Olfr693 and Olfr31 in the dual-luciferase assay.** ‘+’ or ‘–‘ in each column indicates if the olfactory receptor responded or had no effect to that specific chemical.
(XLSX)

Table S3 **Mouse Taqman GPCR array data.** List of all the GPCRs screened in this study and their corresponding C_t_ values are listed. The average ΔC_t_ ± SD values are also listed for each GPCR based on the data obtained from four independent runs.
(XLSX)
