# Supplementary figures and images for: Identification and Characterization of Novel Renal Sensory Receptors
Source: PLoS One. 2014 Oct 23;9(10):e111053. doi: 10.1371/journal.pone.0111053 (PMC4207771; doi:10.1371/journal.pone.0111053)

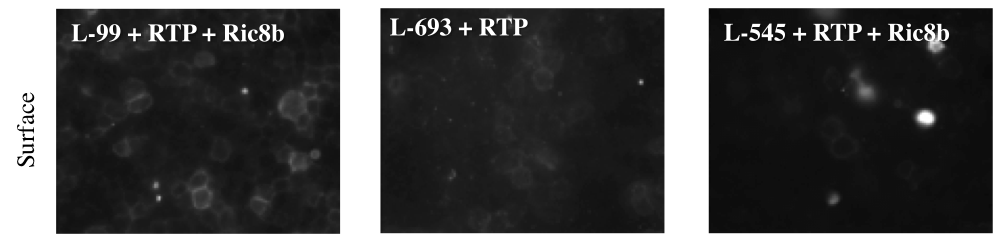

Supplement: Figure S1 — Unenhanced surface images from Figure 4B . Unenhanced images of Olfr99, Olfr693 and Olfr545 in their corresponding conditions that facilitate plasma membrane surface trafficking in HEK293T cells. (TIFF) [file pone.0111053.s001.tiff]
